# Multiple ecological processes underpin the eruptive dynamics of small mammals: House mice in a semi‐arid agricultural environment

**DOI:** 10.1002/ece3.6145

**Published:** 2020-03-05

**Authors:** Peter R. Brown, Anthony D. Arthur, Dean A. Jones, Micah J. Davies, David Grice, Roger P. Pech

**Affiliations:** ^1^ CSIRO Agriculture and Food Canberra ACT Australia; ^2^ Department of Agriculture and Water Resources Canberra ACT Australia; ^3^ CSIRO Land and Water Canberra ACT Australia; ^4^ Manaaki Whenua Landcare Research Lincoln New Zealand; ^5^ Present address: Far North Environmental Consulting Atherton QLD Australia; ^6^ Present address: Batemans Bay NSW 2536 Australia

**Keywords:** abundance, breeding, food addition, mouse plague, survival

## Abstract

1. Mouse plagues are a regular feature of grain‐growing regions, particularly in southern and eastern Australia, yet it is not clear what role various ecological processes play in the eruptive dynamics generating these outbreaks.
2. This research was designed to assess the impact of adding food, water, and cover in all combinations on breeding performance, abundance, and survival of mouse populations on a typical cereal growing farm in northwestern Victoria.
3. Supplementary food, water, and cover were applied in a 2 × 2 × 2 factorial design to 240 m sections of internal fence lines between wheat or barley crops and stubble/pasture fields over an 11‐month period to assess the impact on mouse populations.
4. We confirmed that mice were eating the additional food and were accessing the water provided. We did not generate an outbreak of mice, but there were some significant effects from the experimental treatments. Additional food increased population size twofold and improved apparent survival. Both water and cover improved breeding performance. Food and cover increased apparent survival.
5. Our findings confirm that access to food, water, and cover are necessary for outbreaks, but are not sufficient. There remain additional factors that are important in generating mouse plagues, particularly in a climatically variable agricultural environment.

## Graphical abstract

The ecological processes that underpin the eruptive dynamics of small mammals were manipulated in a semi‐arid agricultural environment through provision of supplementary food, water, and cover to try and generate an outbreak of the feral house mouse. Additional food increased population size twofold and improved apparent survival, both water and cover improved breeding performance, and food and cover increased apparent survival, but we did not generate an outbreak of mice. Our findings confirm that access to food, water, and cover are necessary for outbreaks, but are not sufficient; there remain additional factors that are important in generating mouse plagues, particularly in a climatically variable agricultural environment.

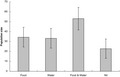

## 1 INTRODUCTION

**Figure 1:**
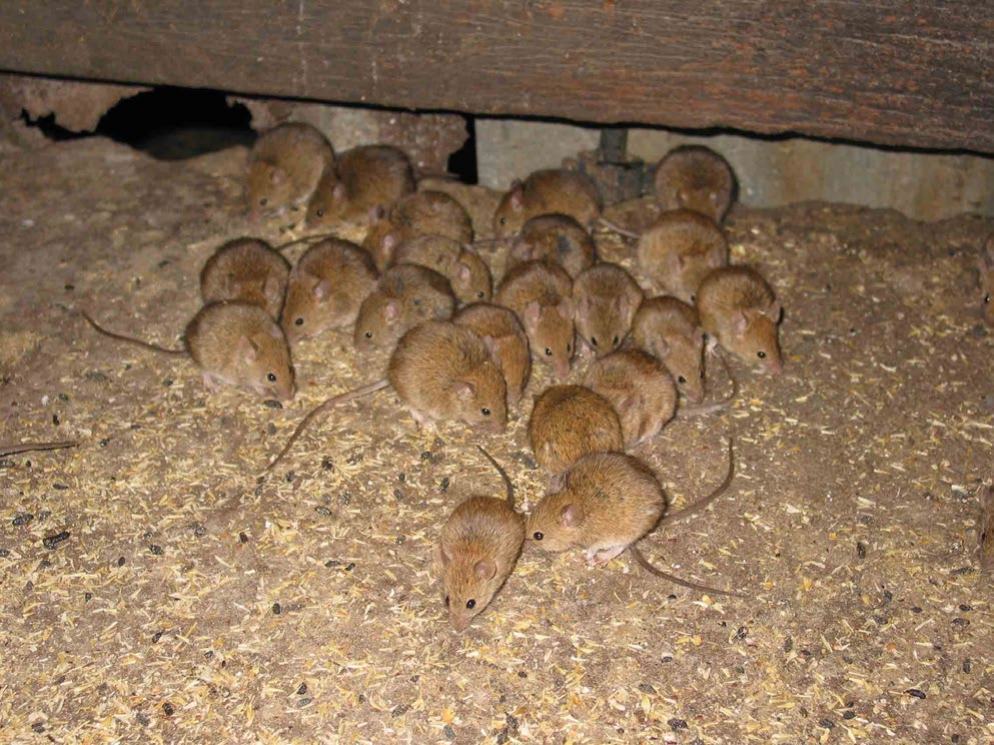
Feral house mice (*Mus musculus domesticus*) can reach very high densities during outbreaks and cause significant damage to grain crops in Australia

Many animal populations are characterized by minor fluctuations in population density or regular population cycles. In contrast, feral house mice (*Mus musculus domesticus*, Figure 1, Gabriel, Stevens, Mathias, & Searle, 2011) in the grain growing, semi‐arid regions of southeastern Australia undergo sporadic eruptions over thousands of square kilometers, where populations increase rapidly from typically low densities of <50 ha^‐1^ to >800 ha^‐1^ (Korpimäki, Brown, Jacob, & Pech, 2004; Singleton et al., 2005). Rainfall during winter and spring seems to be a key driver (Brown & Singleton, 1999; Kenney et al., 2003; Krebs et al., 2004; Pech et al., 1999), but the mechanisms by which rainfall or other processes promotes mouse outbreaks is not well understood.

One possibility is that rainfall increases the availability of high‐quality food which promotes reproduction (Bomford, 1987a, 1987b; Singleton, Krebs, Davis, Chambers, & Brown, 2001; White, 2002). However, the results of food addition experiments have been conflicting. In a recent meta‐analysis of the effects of food supply and predation during 148 experiments, Prevedello, Dickman, Vieira, and Vieira (2013) found that food supplementation increased small mammal population densities 1.5‐fold and that immigration was the major reason for this in open populations. There were no effects on survival, although increases in reproductive rate were detected, but were minor compared to immigration (Prevedello et al., 2013). In specific studies effects on reproduction have been variable. In Australia, supplementary food resulted in an increase in the proportion of females breeding and an extension of the breeding season (Bomford & Redhead, 1987), but supplementary food had no effect on breeding performance during a year when populations increased to high numbers even in the absence of supplementary food (Jacob, Hinds, Singleton, Sutherland, & Ylönen, 2007; Ylönen, Jacob, Runcie, & Singleton, 2003). In the latter study, the failure to increase breeding may have occurred because insufficient free water was available to exploit the high protein content of the dry supplementary food (Ylönen et al., 2003). In a replicated experiment preceding the one reported in this paper, the addition of supplementary food and water had no biologically meaningful effect on population size or breeding performance during a year when populations density remained low (Brown, Arthur, Jones, & Davies, 2008).

Other ecological factors which have been considered in limiting mouse populations in Australia in years when outbreaks do not occur include predation (Kay, Twigg, Korn, & Nicol, 1994; Sinclair, Olsen, & Redhead, 1990), disease (Singleton et al., 2005), and social regulation (Krebs, Chitty, Singleton, & Boonstra, 1995; Sutherland & Singleton, 2006; Sutherland, Spencer, Singleton, & Taylor, 2005). Predators of mice in agricultural areas in southeastern Australia include foxes (*Vulpes vulpes*), feral cats (*Felis catus*), raptors such as brown falcons (*Falco berigora*), black‐shouldered kites (*Elanus axillaris*) and Australian kestrels (*Falco cenchroides*), barn owls (*Tyto alba*), and various species of snakes. Under seminatural conditions protection from predation using artificial cover has been shown to reduce both the lethal and nonlethal impacts of predators on house mice (Arthur, Pech, & Dickman, 2004, 2005). In that experiment mice in both predator exclusion enclosures and those provided with artificial cover began breeding earlier in spring than those that had limited protection from predators (Arthur, Pech, & Dickman, 2004). An early onset of breeding is characteristic of mouse population outbreaks in southeastern Australia, with breeding in an outbreak year usually commencing earlier in southern hemisphere spring, in the middle of August, compared with late September–early October in other years (Singleton et al., 2001).

In addition to a rapid population increase in spring from low numbers, outbreaks in this study area follow one of two patterns (Singleton et al., 2001, 2005). In some cases, numbers decline rapidly in autumn and remain low over the following spring/summer period, while in others numbers decline less rapidly and populations increase again the following spring. These two‐year outbreaks cause considerable damage because they result in very high densities of mice from the early stages of crop development right through crop maturation (Brown, Huth, Banks, & Singleton, 2007; Caughley, Monamy, & Heiden, 1994). It is currently not known why these different patterns occur.

This study followed on from that of Brown et al. (2008) using as experimental units many of the same plots placed along fence lines in cereal production areas in the Victorian Mallee. From midwinter (July) 2004 to late winter (August) 2004, the same supplementary high‐quality food and water treatments as the preceding study were still in place. In August 2004, we added artificial cover to half the plots to provide mice with protection from avian and mammalian predators. The cover remained in place until the completion of the study in winter (June) 2005, giving us a 2 × 2 × 2 factorial design. We assessed treatment effects on the proportion of adult females breeding, overall population numbers, and the apparent survival rates of mice. We tested the following predictions.

1. If the absence of high‐quality food or water, or high predation pressure, either alone or in combination was restricting the onset of breeding, then the appropriate combination of treatments (addition of food and/or water and/or cover) would result in an earlier onset of breeding by mice, or a higher proportion of adult females breeding earlier in the breeding season.
2. If the absence of high‐quality food or water or high predation pressure, either alone or in combination was limiting mouse population size, then the appropriate combination of treatments would result in higher population sizes being reached.
3. If the absence of high‐quality food or water or high predation pressure, either alone or in combination was limiting apparent survival of mice, then the appropriate addition of these factors would result in higher apparent survival rates.

## 2 METHODS

### 2.1 Study site and experimental design

The study was conducted on a grain and sheep property 3 km west of the Mallee Research Station at Walpeup, Victoria, Australia (35°08′S, 142°02′E) between winter (July) 2004 and winter (June) 2005. The area has an average yearly rainfall of 335 mm. More rain falls in winter when rainfall is less variable (May–August, mean = 130 mm CV = 39%) than in summer (January–April, mean = 89 mm CV = 63%). The other four months (September–December) have on average 118 mm falls with a CV of 50%. Rainfall records for during and preceding the study were obtained from the Mallee Research Station and used to predict the likely background numbers of mice based on current models (Kenney et al., 2003). The farm was approximately 2,000 ha in size, and each year about 50% of the fields are planted with wheat or barley. The remaining 50% of the fields are pastures grazed by sheep. Typical pasture consists of the wheat and barley stubble remaining from the previous year's harvest.

**Figure 2:**
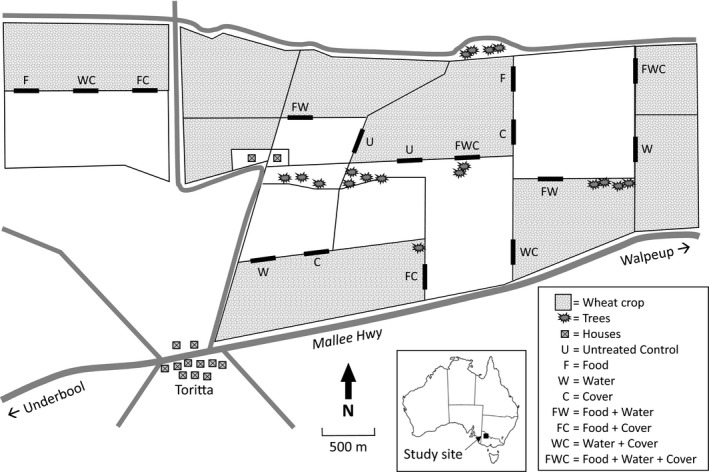
Layout of study site between Walpeup and Toritta, northwestern Victoria. There were 16 experimental sites established, each 240 m in length. There were two replicates of each treatment (untreated control, food, water, cover, food + water, food + cover, water + cover, and food + water+cover), which were assigned randomly. Sites were established along internal fence lines between a wheat crop (shaded) and pasture for sheep grazing (nonshaded)

Plots (experimental units) were 240 m sections of internal boundary fences with winter cereal on one side (barley or wheat) and pasture (normally grazed) on the other (Figure 2). Twelve of our 16 plots were the same as those used in Brown et al., (2008), but four plots had to be moved to maintain cereal crop on one side and pasture on the other, because of crop rotation at our study site. For the first period of the study (July 2004–August 2004; 42 days), our treatments were additional food and additional water in a 2 × 2 factorial design, that is, four replicates of each combination. In spring (August) 2004, we randomly added cover to two of the four of each combination to give a 2 × 2 × 2 factorial design (Figure 2), that is, two plots were provided with additional food, water, and cover; two with additional food and cover; two with additional water and cover; two with additional cover only; two with additional food and water; two with additional food only; two with additional water only; and two were experimental control plots. These treatments then remained in place until the end of the experiment in June 2005. Each plot was separated from others by at least 300 m.

### 2.2 Experimental treatments

**Figure 3:**
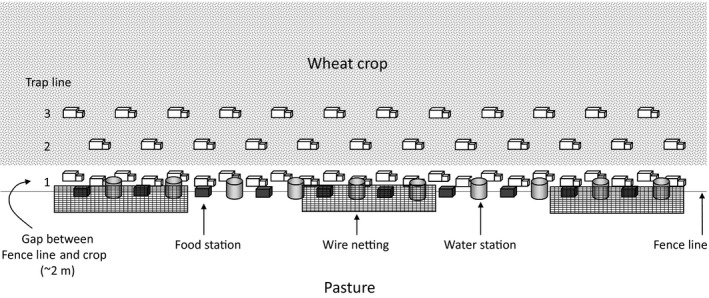
Schematic representation of a 240 m experimental site showing the approximate location of the three trap lines, the food and water containers and the wire netting (three sections of 40 m) along a fence line between a wheat crop and a pasture crop used for sheep grazing. Three additional water stations (20 L drums) were provided (not shown). For sites without supplementary food or water, empty food and water containers were provided. On sites without supplementary cover, no wire netting (cover) was provided

The basic layout of each experimental treatment is shown in Figure 3. The additional food was pelletized rat/mouse food (Rat and Mouse Breeder Pellets, Gordon's Specialty Stockfeeds, Yanderra, Australia) with a minimum crude protein content of 23%, minimum crude fat 6%, and maximum crude fiber 5%. At least one kilogram of food was added to each of ten five‐liter plastic containers. Up to 2.5 kg of food was added to frequently visited food stations. Containers were spaced evenly along the 240 m length of each plot (one every 20 m) and tied to the bottom of the fence using tie wire. At each trapping session, the amount of food removed was recorded (based on change in weight from previous session) and additional food was added when necessary.

Additional water was provided in 20 L plastic containers. A laboratory water nozzle (AHS 25, 65 mm, CF Maddock and Company) was fitted to a rubber stopper that was placed in a hole cut approximately 50 mm from the bottom of the container. Wild mice under laboratory conditions are known to learn the technique of using the nozzles quickly, and we had evidence from the preceding experiment that mice in the field would use the water (Brown et al., 2008). Water containers were spaced evenly every 20 m midway between the food stations (Figure 3). At each trapping session, the nozzles on all water containers were tested by hand for proper function to ensure they had not become clogged and the water levels were checked and maintained to at least 10 L (half capacity). From October 2004 to the completion of the experiment, three additional 20‐L drums of water were placed in fixed locations along water treatment plots, approximately 40 m from each end of the plot and one in the middle. These were added to provide animals with another source of water, supplementing the existing method of water provision. These water stations were buried so the tops of drums were at ground level. There was a 60 mm opening on the top of each drum. Secured to the opening was a 60 cm length of 15 mm nylon rope that was placed into the drum to provide climbing access to and from the available water. These stations were covered with a 1 m^2^ sheet of corrugated iron to reduce sand and dust contaminating water. All additional water drums were filled to at least two‐third full. The water level of additional drums was monitored during every session and replenished when necessary. Additional water stations were always covered by the existing wire mesh (described below) if they were on cover treatment plots.

In order to test whether mice were drinking from the water stations, a fluorescent nontoxic, flavorless, and odorless xanthene dye (Rhodamine B) was added to the water (0.2 g/L) on several plots where mouse densities were highest. This included either or both the above ground and or the buried water stations. If mice were drinking the water, Rhodamine B would appear in the blood up to three days after drinking or in the whiskers for up to seven weeks after drinking (Jacob, Jones, & Singleton, 2002; Fisher, 1999). On these plots, blood samples and whiskers were collected and analyzed using the methods described in Jacob et al., (2002).

Empty food and water containers were placed at all plots where food or water was not provided to control for any effect of the containers, for example, provision of cover. We anticipated mice would use the containers as shelter to build burrows beneath and that these burrows might lead to some mice monopolizing the shelter, the food, or the water. To avoid this, at each trapping session, containers that had burrows under them were moved up to 3 m along the fence.

Additional cover was provided using Waratah rabbit proof mesh (1.2 m wide, 105/4/1.4 gauge) that was cut into 40 m lengths (Figure 3). Three lengths were placed at each cover plot and fixed length ways along the fence. One length was positioned in the middle of the fence line, and the other two lengths were positioned at either end of the fence line. The wire mesh was secured to the fence approximately 30 cm above ground, and the outer edge was crimped every 2 m by hand so the mesh would remain above ground level and provide adequate cover. On sites without additional cover, no wire netting (cover) was provided.

### 2.3 Live trapping

Mice were live‐trapped using Longworth small mammal traps (24 × 7 × 9 cm, Longworth Scientific) on all plots for three consecutive nights in midwinter (July) 2004, late winter (August) 2004, spring (October) 2004, midsummer (December) 2004, late summer (February) 2005, autumn (April) 2005, and early winter (June) 2005. Forty‐eight traps were set in three parallel lines along the 240 m length of each plot for each trapping session (Figure 3). In line 1, 24 traps were set with 10 m spacing between traps in the grassy margin within 1 m of the fence. Line 2 consisted of 12 traps set 10 m into the crop from the fence with 20 m spacing between traps. Line 3 consisted of 12 traps set 20 m into the crop from the fence with 20 m spacing between traps. The starting point of lines 2 and 3 was offset by 10 m. On first capture animals were individually marked using microchips (Allflex Pty Ltd, 11.5 × 2.1 mm, FDX‐B microchip) and an ear punch specific to that trapping session. Sex, breeding status, body length, and weight were recorded on initial capture of individuals in each trapping session (Brown et al., 2008).

### 2.4 Effect of treatments on reproduction

We assessed the effect of treatments on the proportion of adult females >71 mm in length that were in breeding condition (evidence of lactation or pregnant as determined by palpation). This length threshold is based on previous studies of house mice, which show that females can become sexually mature at this length (Singleton, 1983). We used generalized linear modeling with binomial errors. Adjusted Akaike information criterion (AIC_c_) was calculated from the minimized negative log‐likelihood using standard formulas (Burnham & Anderson, 2002). The weight of support for each model conditional on both the data and all models in the set was calculated as described in Burnham and Anderson (2002). Analyses were carried out in program R (R Development Core Team, 2007).

### 2.5 Effect of treatments on population size

Population size was estimated using the Jackknife estimator in Program Capture (Otis, Burnham, White, & Anderson, 1978). The Jackknife estimator, which allows for individual heterogeneity in capture probability, may be biased low for estimating abundance of house mice, but still performs well as an index of abundance and hence is appropriate for comparing changes in population size under the treatments (Davis, Akison, Farroway, Singleton, & Leslie, 2003). There was no evidence of correlation structure between successive measurements and results clearly indicated no treatment effects in the early stages of the experiment, so the effect of treatment on mouse abundance was analyzed separately for each trapping session. We used generalized linear modeling with normal errors. Residual plots indicated that these were appropriate and data transformation was not required. Adjusted Akaike information criterion (AIC_c_) was calculated from the minimized negative log‐likelihood, and the weight of support for each model conditional on both the data and all models in the set was used for inference (Burnham & Anderson, 2002). Akaike weights were used to calculate model‐averaged parameter estimates and model average standard errors. These estimates incorporate uncertainty from both individual models and the relative support of each model in the set (Burnham & Anderson, 2002).

### 2.6 Effect of treatments on apparent survival

Mark‐recapture modeling of individually marked mice was used to assess treatment effects on apparent survival (Φ), which includes actual survival and permanent emigration. The winter (August) 2004 session was used as the starting point for survival analyses because this was the time when all treatments including the cover treatment were in place. Program U‐CARE was used to test the goodness of fit (GOF) of a simplified starting model with two “age‐classes” for Φ and with time dependence in both age‐classes (Choquet, Reboulet, Lebreton, Gimenez, & Pradel, 2005). The “age‐classes” divide mice into those previously marked and those marked for the first time at a particular trapping session. The former tended to be adult animals, while the latter was a mix of juveniles, transient adults, and resident adults that escaped capture on previous occasions (Arthur, Pech, & Dickman, 2005). Insufficient data were available to define age‐classes based on size.

A highly significant ($\chi_{4}^{2}$ = 34.6, *p* < .001) Test 3.sr in U‐CARE (Choquet et al., 2005) indicated different apparent survival between newly marked animals and already marked animals. Combining tests 3.sm, 2.ct, and 2.cl in U‐CARE indicated that a model with 2 “age‐classes” for Φ and time‐varying capture probability (p) was a good starting point for model selection. This model produced a $\chi_{8}^{2}$ value of 7.8 (*p* = .45) indicating there was no need to adjust for overdispersion (Choquet et al., 2005). Mark‐recapture modeling was then conducted using the program R (R Core Development Team, 2007) package RMark (Laake & Rexstad, 2008) as an interface for Program MARK (White & Burnham, 1999). Adjusted Akaike criteria were used to calculate Akaike weights. These combined with model‐averaged parameters estimates were used for inference (Burnham & Anderson, 2002). The best model that did not include different apparent survival for newly marked and previously marked animals had a ΔAIC_c_ of 18.02, consistent with the results of the U‐CARE test.

### 2.7 Biomass and grain samples

To measure availability of natural food and cover, biomass and grain samples were collected using the same techniques as described by Brown et al., (2008). Biomass samples were estimated from 0.25 m^2^ quadrats (*n* = 20 per site) using a modified comparative yield technique (Friedel & Bastin, 1988; Haydock & Shaw, 1975). Reference photographs were used to measure cover and relative background food availability for mice from the crop, and from the area of grasses and weeds between the crop and fence line. Random samples of grain (*n* = 10 per site) were collected from 0.1 m^2^ quadrats immediately after harvesting in December 2004 and again six months after harvest in June 2005.

## 3 RESULTS

### 3.1 Rainfall

**Figure 4:**
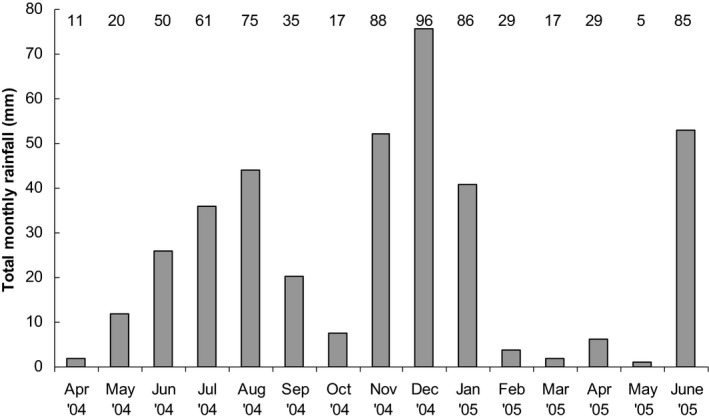
Rainfall records from the Mallee Research Station spanning the experiment. The number above the column shows the percentage of rainfall records for the month from the last 91 years that fall below the value observed during the study

Based on the logistic regression model of Kenney et al. (2003), the probability of an outbreak from the rainfall that spanned our experiment (Figure 4) was 0.45 (“moderate” to “high”) given the relatively good rainfall through summer (November 2004, December 2004, and January 2005). Using the “early predictor” model of Kenney et al. (2003), the maximum autumn abundance was predicted to be an adjusted trap success (ATS) of 61 which would be considered a small outbreak (Singleton et al., 2005). Using the “late predictor” model of Kenney et al. (2003), the maximum autumn abundance was predicted to be ATS of 15. These predictions suggest relatively good conditions for mice during our experiment, at least in the early stages of the experiment, that is, spring 2004.

### 3.2 Use of food and water

On plots provided with supplementary food throughout the experiment consumption of food between consecutive sessions ranged from a minimum of 96 g station^−1^ plot^−1^ to a maximum of 1,359 g station^−1^ plot^−1^, indicating that mice used the supplementary food at all times. Average consumption per station per plot was 395 ± 140 (*SE*) g in August 2004, 357 ± 120 g in October 2004, 241 ± 81 g in December 2004, 395 ± 130 g in February 2005, 803 ± 185 g in April 2005, and 994 ± 209 g in June 2005. In December 2004, immediately after harvest, there was on average 72.2 ± 8.7 g/m^2^ of spilt grain (equivalent of 722 kg/ha); then, in June 2005 there was on average 9.6 ± 2.1 g/m^2^ of spilt grain (equivalent of 96 kg/ha; a reduction of 87% over 6 months).

Mice were drinking from the water containers. In late winter (August) 2004 and spring (October) 2004 when water was provided in drums fitted with nozzles five and nine mice, respectively, had their whiskers checked and none contained Rhodamine B. In late summer (February) 2005, five of 16 mice had Rhodamine B in their whiskers (additional water had been provided in below ground open drums since October). Two of six mice tested had detectable levels of Rhodamine B in their blood. Eight and six of the trapped mice in February and June, respectively, also had obvious signs of Rhodamine B on their fur (from numerous mice). In April 2005, eight of the trapped mice had obvious signs of Rhodamine B in their scats (from numerous mice).

There was significantly more plant biomass in the crop (1,736 ± 47 kg/ha) than along the fence lines (1,432 ± 32 kg/ha; *t*_96_ = 4.97, *p* < .0001). Biomass varied significantly over time for both fence biomass (*F*_5,48_ = 32.08, *p* < .0001) and crop biomass (*F*_5,48_ = 59.58, *p* < .0001). Biomass was highest along fence lines in August 2004 (1,990 kg/ha) and steadily declined until June 2005 (1,162 kg/ha). The biomass in crop was lowest in August 2004 (908 kg/ha), but increased in October 2004 (2,366 kg/ha) as the crop matured, then steadily declined through to June 2005 (1,629 kg/ha). Fence line biomass varied between treatments (*F*_7,48_ = 2.30, *p* < .05) with the water treatment having slightly higher biomass (1,598 ± 603 kg/ha) than other treatments. There was no treatment effect for crop biomass. No interactions were significant.

### 3.3 Predator activity

Signs of fox (*Vulpes vulpes*) activity including tracks in the sandy soil, scats, and digging were observed around all sites during all trapping sessions. On a few occasions foxes disturbed traps. Raptors including brown falcon (*Falco berigora*), Australian kestrel (*Falco cenchroides*), and black‐shouldered kites (*Elanus axillaris*) were observed in the study area during all trapping sessions. There were also signs of snake activity including tracks and visual observations. Based on ~30 years experience working on mice in the area, predator levels were typically low as found in nonoutbreak years.

### 3.4 Effect of treatments on breeding

Pregnant and/or lactating females were caught in August 2004 across all treatments, and hence, breeding continued through winter (Brown et al., 2008). Up to 6 adult females were caught on plots in this trapping session (*n* = 43 in total), representing 37% (±7) of all adult females captured showing signs of breeding. There was no evidence that the proportion of adult females breeding was influenced by the water or food treatments imposed since July 2004, with an intercept‐only model being the second‐ranked model with an Akaike weight of 0.20.

Between one and 12 adult females were caught on plots in spring (October) 2004 (*n* = 79 in total). The top six ranked models with a combined Akaike weight of 0.94 all included the cover treatment. Based on the top‐ranked model (Table 1), 67% (±7, *n* = 46) of adult females were breeding on plots with added cover and 30% (±8, *n* = 33) of adult females were breeding on plots without artificial cover. Based on the second‐ranked model, 75% (±7) of females were breeding on plots with water and added cover, 41% (±11) were breeding on plots with water but no added cover, 56% (±10) were breeding on plots with cover but not water and 23% (±8) were breeding on plots without water or cover treatments. At this time, trapped adult females were larger on plots with added cover and water than on other plots (mixed‐effects model water:cover interaction term *t*_12_ = 2.4, *p* = .03; cover and water length = 86.5 ± 1.1 mm, water only = 79.6 ± 1.6 mm, cover only = 82.8 ± 1.4 mm; neither treatment = 82.5 ± 1.4 mm).

**Table 1:** Model selection table for the proportion of adult females breeding in spring (October) 2004 and late summer (February) 2005 showing all possible treatment combinations (supplementary food; supplementary water; artificial cover) including additive and multiplicative effects

Session	Model	*N*	AIC_c_	ΔAIC_c_	ω*_i_*
October 2004	Cover	2	50.4	0.0	0.34
	Water + Cover	3	50.4	0.0	0.33
	Food + Cover	3	52.8	2.4	0.10
	Water*Cover	4	53.6	3.2	0.07
	Food + Water+Cover	4	53.6	3.2	0.07
	Food*Cover	4	55.6	5.2	0.02
	Water	2	56.6	6.3	0.01
	Food*Cover + Water	5	57.4	7.0	0.01
	Water*Cover + Food	5	57.5	7.1	0.01
	Food*Water + Cover	5	57.8	7.5	0.01
	Food + Water	3	58.2	7.8	0.01
	Null	1	58.6	8.2	0.01
	Food	2	59.2	8.8	0.00
	Food*Water	4	61.8	11.4	0.00
	Food*Water*Cover	8	77.1	26.7	0.00
February 2005	Food*Water	4	62.4	0.0	0.66
	Food*Water + Cover	5	65.0	2.6	0.18
	Water*Cover	4	67.7	5.4	0.04
	Water	2	67.8	5.4	0.04
	Water + Cover	3	68.4	6.1	0.03
	Null	1	70.2	7.8	0.01
	Food + Water	3	71.0	8.6	0.01
	Cover	2	71.5	9.2	0.01
	Food + Water+Cover	4	72.3	9.9	0.00
	Water*Cover + Food	5	72.3	10.0	0.00
	Food*Cover + Water	5	72.8	10.5	0.00
	Food	2	72.9	10.5	0.00
	Food + Cover	3	74.7	12.4	0.00
	Food*Cover	4	75.6	13.2	0.00
	Food*Water*Cover	8	78.7	16.3	0.00

Note

The Null model is an intercept‐only model where the proportion breeding is the same in all plots.

Abbreviations: AIC_c_, Akaike information criterion adjusted for sample size; ΔAIC_c_, the difference between the model and the best model; *N*, number of parameters; ω*_i_*, Akaike weight.

There was no strong support for any treatment effects on the proportion of adult females breeding in midsummer (December) 2004, autumn (April) 2005, or midwinter (June) 2005 when 70% (±6), 49% (±5), and 30% (±4), respectively, of all adult females caught were showing signs of breeding. In late summer (February) 2005, up to 11 adult females were caught on plots (*n* = 87 in total). In June 2005, 75% of those showing signs of breeding were lactating and 25% were pregnant. The highest ranked model had approximately three times the support of the second‐ranked model and suggested a food by water interaction (Table 1), with 27% (±8, *n* = 30) of adult females showing signs of breeding on plots with food and water, 88% (±8, *n* = 16) breeding on plots with food but not water, 54% (±10, *n* = 24) breeding on plots with water but not food, and 41% (±12, *n* = 17) breeding on plots without food or water treatments. The average length of adult females caught at this time did not vary by treatment.

### 3.5 Effects of treatments on population size

**Figure 5:**
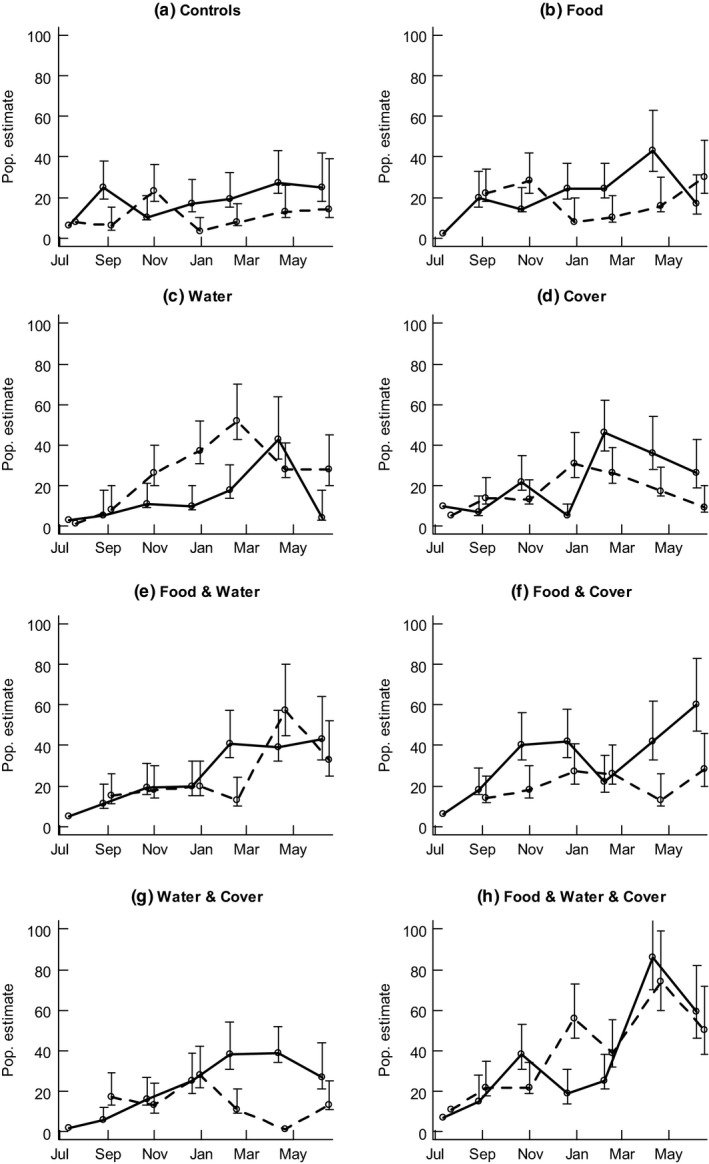
Mouse population changes throughout the experiment on the 16 plots (both replicates shown for each treatment). Population estimate based on the mark‐recapture Jackknife estimator. Error bars are 95% confidence intervals

**Figure 6:**
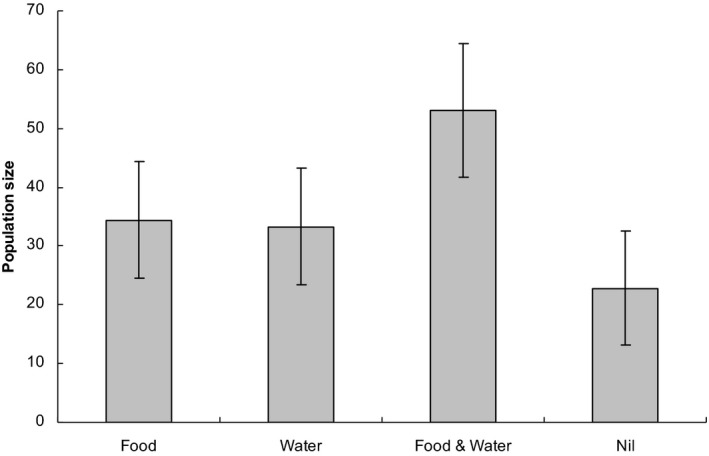
Model‐averaged estimates (±*SE*) of population size in response to treatments in autumn (April) 2005, based on the top 5 models (combined Akaike weight 0.85) shown in Table 2, that is, the 5th ranked null model is included in the estimates

From August 2004 through to February 2005, there were no clear effects of treatment on population size (Figure 5), with the null model receiving strong support in all sessions. In autumn (April) 2005, models including food and/or water effects were ranked in the top 4 (Table 2). Populations were larger on plots with both the food and water treatment in combination compared with plots where only one or the other was provided (Figure 6). There was weak evidence that food or water in isolation resulted in higher numbers compared with plots where neither was present (Figure 6).

**Table 2:** Model selection table for the effects of treatments on population size in autumn (April) and midwinter (June) 2005 showing all possible treatment combinations including additive and multiplicative effects

Session	Model	*N*	AIC_c_	ΔAIC_c_	ω*_i_*
April 2005	Food + Water	4	146.9	0.0	0.28
	Food*Water	5	147.3	0.4	0.22
	Food	3	148.2	1.3	0.14
	Water	3	148.6	1.6	0.12
	Null	2	149.2	2.3	0.09
	Food + Water + Cover	5	150.9	4.0	0.04
	Food + Cover	4	151.6	4.7	0.03
	Water + Cover	4	151.9	5.0	0.02
	Cover	3	152.1	5.1	0.02
	Food*Water + Cover	6	152.2	5.2	0.02
	Food*Cover + Water	6	154.7	7.8	0.01
	Food*Cover	5	154.9	8.0	0.01
	Water*Cover + Food	6	156.1	9.2	0.00
	Water*Cover	5	156.2	9.3	0.00
	Food*Water*Cover	9	171.7	24.8	0.00
June 2005	Food	3	132.9	0.0	0.38
	Food + Cover	4	133.7	0.7	0.26
	Food*Cover	5	135.3	2.3	0.12
	Food + Water	4	135.5	2.6	0.10
	Food + Water+Cover	5	136.8	3.9	0.06
	Food*Water	5	138.6	5.7	0.02
	Food*Cover + Water	6	139.1	6.2	0.02
	Null	2	139.4	6.5	0.01
	Food*Water + Cover	6	140.5	7.6	0.01
	Cover	3	141.0	8.1	0.01
	Water	3	142.0	9.0	0.00
	Water*Cover + Food	6	142.1	9.2	0.00
	Water + Cover	4	144.0	11.1	0.00
	Water*Cover	5	148.4	15.5	0.00
	Food*Water*Cover	9	163.5	30.6	0.00

Note

The null model is an intercept‐only model where the proportion breeding is the same in all plots.

Abbreviations: AIC_c_, Akaike information criterion adjusted for sample size; ΔAIC_c_, the difference between the model and the best model; *N*, number of parameters; ω*_i_*, Akaike weight.

**Figure 7:**
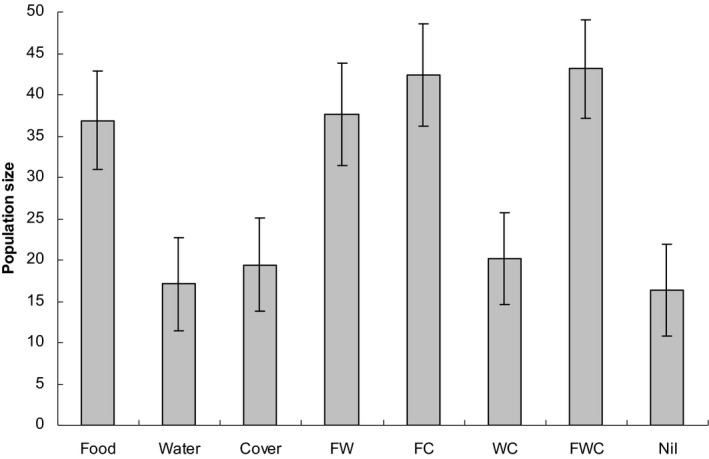
Model‐averaged estimates (±*SE*) of population size in response to treatments in midwinter (June) 2005, based on the top four models (combined Akaike weight 0.87) shown in Table 2. Treatment combinations are supplementary food and water (FW), food and added cover (FC): water and cover (WC), and food, water, and cover (FWC)

In midwinter (June) 2005, the top seven models, with a combined weight of 0.96, included an effect of food (Table 2). Populations were approximately two times larger on plots with added food compared to plots without added food (Figure 7). There was weak evidence for a small additive effect of the cover treatment; plots with added cover appeared to have slightly more mice than plots with equivalent underlying other treatments without added cover (Figure 7).

### 3.6 Effects of treatments on apparent survival

**Figure 8:**
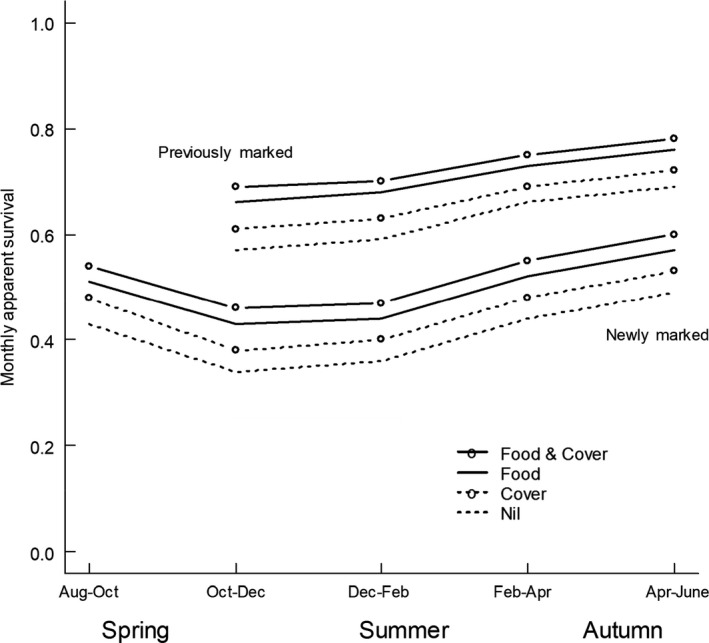
Model‐averaged estimates of apparent survival from the top 10 models in Table 3. The water treatment made no biologically significant difference to apparent survival rates, so it has been removed from the figure to aid clarity. For example, the model‐averaged predictions for the survival rates under the food and cover and water treatment were essentially the same as those for the food and cover treatment

The top 18 models (collective weight = 0.924) and 23 of the top 25 models included an effect of food on apparent survival, with a collective model weight of 0.958 of a possible 0.972 (Table 3). There was no support of models where capture probability varied by plot. In the top 25 models, the collective model weight for models including added cover was 0.654 and for models including water was 0.371 (Table 3). Models with time‐varying effects of treatments did not fall in the top 25. Model‐averaged estimates indicated the following underlying patterns in apparent survival (Figure 8); previously marked animals had higher apparent survival rates than newly marked animals (which include a high proportion of younger animals) throughout the experiment. All animals were considered newly marked in August 2004, and the apparent survival rate during spring (i.e., between August and October) 2004 showed an intermediate pattern, consistent with this comprising a higher proportion of older animals in the “newly marked” group at this time. There was evidence that food and cover increased the apparent survival rate of newly marked and previously marked animals throughout the experiment, with food having the larger effect (Figure 8).

**Table 3:** Model selection table for the effects of treatments on apparent survival (Φ)

Rank	Model	*N*	AICc	ΔAICc	ω*_i_*
1	Φ(~a2 + time + food + cover)p(.)	9	1,384.17	0.00	0.15
2	Φ(~a2*food)p(time)	9	1,384.82	0.65	0.11
3	Φ(~a2 + time + food)p(.)	8	1,385.14	0.97	0.09
4	Φ(~a2 + food*cover)p(time)	10	1,385.40	1.23	0.08
5	Φ(~a2 + time + food + water*cover)p(.)	11	1,385.74	1.57	0.07
6	Φ(~a2 + time + food*cover)p(.)	10	1,385.76	1.59	0.07
7	Φ(~a2 + food + water + cover)p(time)	10	1,385.95	1.78	0.06
8	Φ(~a2 + food)p(time)	8	1,386.10	1.93	0.06
9	Φ(~a2 + time + food + water + cover)p(.)	10	1,386.16	1.99	0.05
10	Φ(~a2 + time + food + water)p(.)	9	1,387.05	2.88	0.04
11	Φ(~a2 + time +food + cover)p(time)	13	1,387.26	3.09	0.03
12	Φ(~a2 + food*water + cover)p(time)	11	1,387.40	3.23	0.03
13	Φ(~a2 + time + food*cover + water)p(.)	11	1,387.76	3.59	0.02
14	Φ(~a2 + time + food*water + cover)p(.)	11	1,388.02	3.85	0.02
15	Φ(~a2 + time + food + water*cover)p(time)	15	1,388.30	4.13	0.02
16	Φ(~a2 + food*water*cover)p(time)	14	1,388.46	4.29	0.02
17	Φ(~a2 + time + food*cover)p(time)	14	1,388.92	4.75	0.01
18	Φ(~a2 + time + food*water)p(.)	10	1,388.99	4.82	0.01
19	Φ(~a2 + time + cover)p(.)	8	1,389.69	5.52	0.01
20	Φ(~a2 + food*water)p(time)	10	1,389.74	5.57	0.01
21	Φ(~a2 + time + food*water*cover)p(.)	14	1,389.94	5.77	0.01
22	Φ(~a2 + time + food*water + cover)p(time)	15	1,390.52	6.35	0.01
23	Φ(~a2 + time + food*cover + water)p(time)	15	1,390.89	6.72	0.01
24	Φ(~a2 + time + food + water)p(time)	13	1,390.90	6.73	0.01
25	Φ(~a2 + time)p(.)	7	1,391.02	6.85	0.01
..	..	..	..	..	..
207	Φ(~a2 + food + cover)p(time)	9	7,360.79	5,976.62	0.000

Abbreviations: AIC_c_, Akaike information criterion adjusted for sample size; ΔAIC_c_, the difference between the model and the best model; *N*, number of parameters; ω_i_, Akaike weight; a2, two “age‐classes”—newly marked and previously marked; p(.), constant capture probability.

## 4 DISCUSSION

We were unable to generate an outbreak through provision of food, water, cover, or any combination of these. Our experiment commenced in winter, and during the early stages, background conditions were such that the early predictor model of Kenney et al. (2003) indicated the potential for a small outbreak. We did see a twofold increase in population density late in the experiment on sites where additional food was provided, which was slightly higher than the 1.5‐fold increase in population densities found by Prevedello et al. (2013) from a global meta‐analysis of food addition experiments. While we did not generate an outbreak, some of the responses to our treatments were consistent with demographic patterns associated with outbreaks which included treatment effects on both reproduction and apparent survival.

Mouse population eruptions in southeastern Australia are characterized by an early onset of breeding (Brown & Singleton, 1999; Kenney et al., 2003; Krebs et al., 2004; Singleton et al., 2001), and we found that breeding continued over winter and spring in 2004 on many of our plots regardless of treatment. It has been suggested that early breeding occurs when pasture growth and seed set promotes it (Singleton, 1989; Mutze, Veitch, & Miller, 1990; Pech et al., 1999) and our measurements were consistent with this; we found high levels of biomass of grasses and weeds along the area between the crop and fence lines early in the experiment. However, despite the apparent importance of food, Jacob et al. (2007) found that food addition did not alter ovulation rates or litter size. We also found no effect of food addition alone on early breeding, both in this experiment and in our preceding one (Brown et al., 2008). Adding water alone also had no effect on early breeding. However, our cover treatment appeared to further increase the proportion of females breeding in spring.

In a seminatural setting, protection from predation, either through predator exclusion fencing or by providing cover (in the same way it was provided in this experiment), has been shown to decrease the nonlethal impacts of predators on house mice (Arthur et al., 2004). In that study, protected mice had higher growth rates and began breeding earlier in spring when protection from predation made them more willing to access high‐quality supplementary food. Female mice on the water plus cover treatment in our current experiment were larger and also had the highest proportion breeding in spring, consistent with this. The results suggest that even in the presence of natural cover, artificial cover may have reduced the nonlethal impacts of predation, but that this was not sufficient to produce population level increases at the scale of our plots.

As the experiment progressed, background conditions probably became less favorable for mice, based on the “late predictor” model of Kenney et al. (2003). There was evidence that additional food and cover increased the apparent survival rate of newly marked and previously marked animals, with food having the larger effect. Prevedello et al. (2013) found that population effects were larger when predation was reduced and populations were open to immigration, and that immigration was more important than survival.

There was evidence that mice were eating the food (reduction in weight of food containers) and drinking the water (evidence of Rhodamine B in samples from February–June) at treatment plots, but was this enough? There were up to 40 mice on each plot, and there was roughly 400 g eaten per station (10 stations per plot), so up to 4 kg food consumed/plot. This is equivalent of up to 100 g per mouse per monitoring interval (roughly every 2 months), so equivalent of about 1.67 g day^−1^ mouse^−1^ of additional food consumed. Bomford (1985) estimated that an adult mouse eats about 2.5 g of food each day (roughly 10% of body weight), so the additional food provided roughly two‐thirds of the food required for a typical mouse to meet its daily energy requirements. There was also a large amount of food available as the crop matured (grain yield ~ 2,000–3,000 kg/ha), some of which was spilt at harvest time (722 kg/ha), potentially swamping any food addition at the treatment plots.

House mice (*Mus musculus domesticus*) are physiologically well adapted to semi‐arid environments and can survive without freely available water by obtaining water from their food (Fertig & Edmonds, 1969; Moro & Bradshaw, 1999; Mutze, Green, & Newgrain, 1991; Prakash & Ghosh, 1975). During dry summers, breeding can be limited by low water availability (Newsome, 1969). The level of moisture stress can depend on a range of factors including rainfall, availability of food or shelter and the amount of weeds present (Mutze et al., 1991). Providing additional water for a field population of mice in California USA increased population size by about 35% compared with the experimental control site (Newsome, Stendell, & Myers, 1976), suggesting that water was a key limiting factor. Models with time‐varying effects of treatments did not fall in the top 25, possibly because sample sizes were too small to detect time‐varying effects. There was no effect of treatment on population size up until late summer (February) 2005, despite apparent effects of added cover on breeding, and of supplementary food and added cover on survival. However, it is likely that given the good conditions across all treatments, with particularly good rainfall from late spring to midsummer (November 2004–January 2005), mouse populations were increasing across all sites over this period. Then, rainfall was low during February to May, which allowed treatment effects to appear. There was no widespread outbreak or mouse plague in 2005 or 2006; this was at a time when the Millennium drought was affecting most of southern and eastern Australia (Bureau of Meteorology, 2015).

In autumn (April) 2005, populations were higher on plots that had both food and water added. In midwinter (June) 2005, populations were higher on plots that had supplementary food. While local population dynamics on these plots could have generated these results, an alternative explanation is that mice in the landscape settled (via immigration) at those sites where food was available. Either way these results suggest that food could have been limiting at this time and if high‐quality food is available this could help maintain populations over winter and provide the starting population for the second year of a 2‐year outbreak. Higher apparent survival on plots with food might reflect either increased survival because of food or increased site attachment because of food; that is, mice were less inclined to become nomadic if high‐quality food is available.

Mice are responding to multiple ecological processes, and by trying to make favorable conditions (through provision of food, water, and cover), we were unable to generate an outbreak or a mouse plague. There are a range of other intrinsic factors (e.g., social structure, infanticide, sexual maturation, aggression, dispersal, density dependence) and extrinsic factors (e.g., habitat, cover, food, predation, disease, weather) that are likely to be influencing mouse populations. The rapid decline observed in mouse populations (Brown, 2006) is thought to be driven by extrinsic factors (food depletion and disease; Brown, Singleton, Pech, Hinds, & Krebs, 2010) but also via infanticide (see Sutherland et al., 2005).

Food, water, and cover are important, but not sufficient to generate a mouse outbreak. There has been lots of conjecture about the effects of spatial heterogeneity in these ecosystems (Chambers, Singleton, & Wensveen, 1996; Krebs et al., 1995; Redhead, 1982), but these effects have not been quantified sufficiently to understand what is going on with movements of mice. Low recapture rates are typical (Krebs, Singleton, & Kenney, 1994), which suggests movements at local scales could be quite important. In vertebrate populations with multi‐annual fluctuations, changes in social behavior and kin structure have been proposed as a causal mechanism for changes in spacing behavior which results in density fluctuations (Krebs et al., 1995; Sutherland et al., 2005). The maintenance of female kin groups through the preceding winter significantly improved recruitment during the subsequent breeding season and therefore is necessary for generating mouse outbreaks (Sutherland et al., 2005). Stress‐related genes may also play a role in tempering mouse population dynamics because stress genes are known to flow through to granddaughters especially after peak population densities have been reached (Boonstra, 1994).

Ultimately, we want to be able to confidently forecast when mouse populations are likely to cause economic damage and to encourage farmers and other land managers to take appropriate precautions to manage the risks. Our current models are still adequate for this purpose (Kenney et al., 2003; Pech et al., 1999), but improving our understanding of the mechanisms by which mice increase or decrease would add‐value to these models and further improve our forecasting capability.

## CONFLICT OF INTEREST

None declared.

## AUTHORS CONTRIBUTIONS

All authors designed the study and interpreted the results. PRB and ADA conducted the analyses and led the writing of the manuscript.

## Data Availability

The data used for this study are available through the CSIRO Data Access Portal (https://doi.org/10.25919/5d6c6eb7a1433).
